# Bioactive Secondary Metabolites from the Marine Sponge Genus *Agelas*

**DOI:** 10.3390/md15110351

**Published:** 2017-11-08

**Authors:** Huawei Zhang, Menglian Dong, Jianwei Chen, Hong Wang, Karen Tenney, Phillip Crews

**Affiliations:** 1 Department of Pharmaceutical Sciences, Zhejiang University of Technology, Hangzhou 310014, China; baixl2012@163.com (M.D.); cjw983617@zjut.edu.cn (J.C.); hongw@zjut.edu.cn (H.W.); 2 Department of Chemistry & Biochemistry, University of California Santa Cruz, Santa Cruz, CA 95064, USA; ktenney@ucsc.edu (K.T.); pcrews@ucsc.edu (P.C.)

**Keywords:** marine sponge, *Agelas*, secondary metabolite, natural product, bioactivity

## Abstract

The marine sponge genus *Agelas* comprises a rich reservoir of species and natural products with diverse chemical structures and biological properties with potential application in new drug development. This review for the first time summarized secondary metabolites from *Agelas* sponges discovered in the past 47 years together with their bioactive effects.

## 1. Introduction

**Figure 1:**
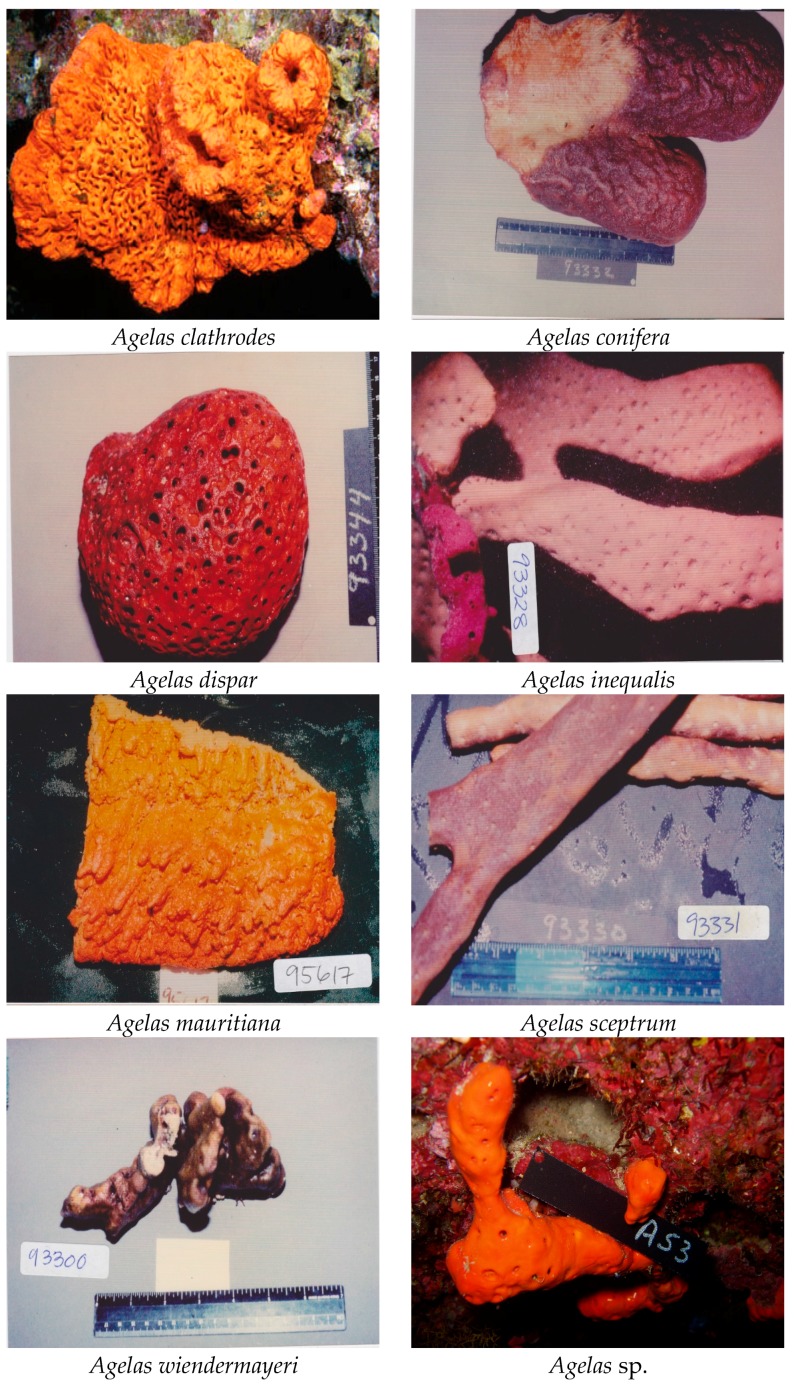
Photos of *Agelas* sponges provided by professor Crews.

The search for natural drug candidates from marine organisms is the eternal impetus to pharmaceutical scientists. For the past six decades, marine sponges have been a prolific and chemically diverse source of natural compounds with potential therapeutic application [1,2]. The marine sponge *Agelas* (Porifera, Demospongiae, Agelasida, Agelasidae) is widely distributed in the marine eco-system and includes at least 19 species (Figure 1): *A. axifera*, *A. cerebrum*, *A. ceylonica*, *A. citrina*, *A. clathrodes*, *A. conifera*, *A. dendromorpha*, *A. dispar*, *A. gracilis*, *A. linnaei*, *A. longissima*, *A. mauritiana*, *A. nakamurai*, *A. nemoechinata*, *A. oroides*, *A. sceptrum*, *A. schmidtii*, *A. sventres*, and *A. wiedenmayeri*. Since the beginning of the 1970s, many research groups around the world have carried out chemical investigation on *Agelas* spp., resulting in fruitful achievements. Their studies revealed that *Agelas* sponges harbor many bioactive secondary metabolites, including alkaloids (especially bromopyrrole derivatives), terpenoids, glycosphingolipids, carotenoids, fatty acids and meroterpenoids [3]. These natural products are an attractive resource for drug candidates due to their rich chemodiversity and interesting biological activities.

## 2. Natural Products from *Agelas* Genus

The chemical diversity of natural products is determined by the biological diversity of organisms. To date, 291 secondary metabolites (**1**–**291**) have been isolated and characterized from the marine sponge *Agelas* spp. (Table 1). These chemicals were introduced and assorted as follows according to their biological sources.

### 2.1. Agelas axifera

**Figure 2:**
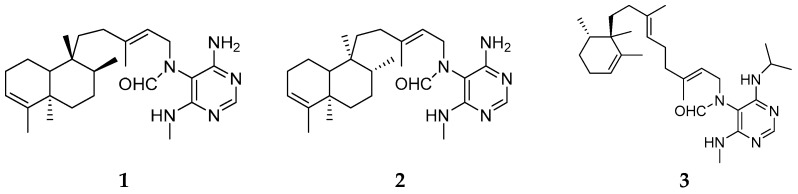
Chemical structures of compounds **1**–**3**.

Three new alkaloids, named axistatins 1 (**1**), 2 (**2**), and 3 (**3**) (Figure 2), were isolated and characterized from *Agelas axifera* collected in the Republic of Palau and found to exhibit inhibitory effects on cancer cell lines, including P388, BXPC-3, MCF-7, SF-268, NCI-H460, KM20L2 and DU-145. The exquisitely sensitive Gram-negative pathogen *Neisseria gonorrheae* and the opportunistic fungus *Cryptococcus neoformans* were inhibited by **1**–**3** with MIC values of 1–8, 2–4, and 8 μg/mL, and 1–4, 2, and 8–16 μg/mL, respectively. Furthermore, these compounds had antimicrobial effect on Gram-positive bacteria, including *Staphylococcus aureus*, *Streptococcus pneumoniae*, *Enterococcus faecalis* and *Micrococcus luteus* [4].

### 2.2. Agelas cerebrum

**Figure 3:**
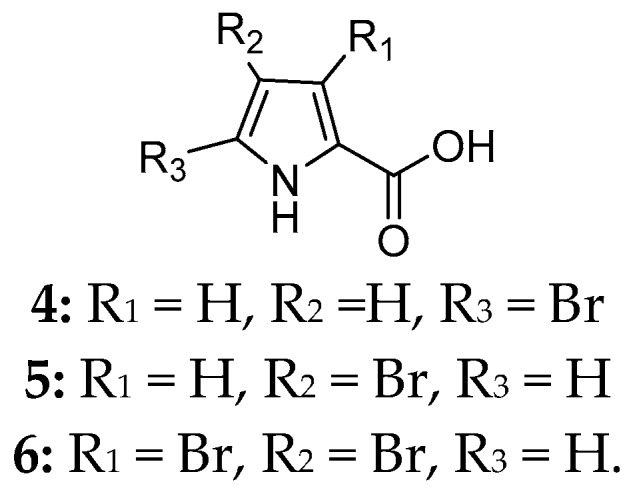
Chemical structures of compounds **4**–**6**.

Marine sponge *Agelas cerebrum* was classified as a new species in 2001 [5]. Chemical investigation of Caribbean specimen *A. cerebrum* led to the isolation of three brominated compounds, 5-bromopyrrole-2-carboxylic acid (**4**), 4-bromopyrrole-2-carboxylic acid (**5**) and 3,4-bromopyrrole-2-carboxylic acid (**6**) (Figure 3) [6]. Biological tests indicated that these isolates had strong cytotoxic activities in vitro against human tumor cell lines at ≥1 mg/mL, including A549, HT29 and MDA-MB-231.

### 2.3. Agelas ceylonica

**Figure 4:**
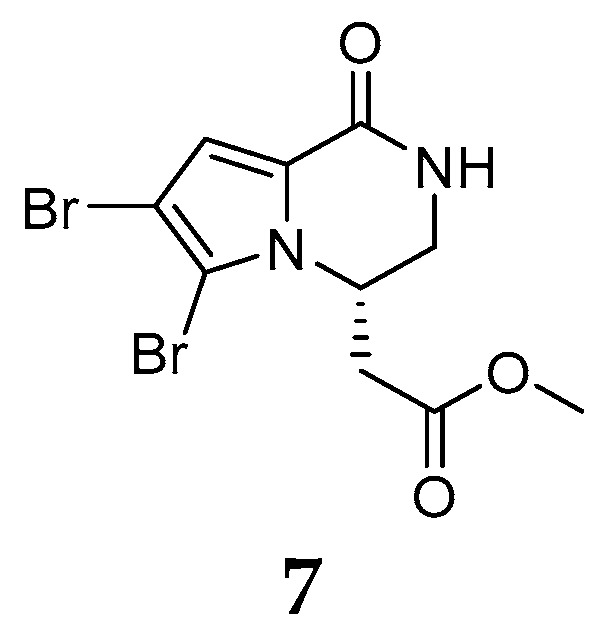
Chemical structures of compounds **7**.

Only one case of chemical study on *Agelas ceylonica* has been reported [7]. The specimen of *A. ceylonica* collected from India Mandapam coast was found to produce one methyl ester hanishin (**7**) (Figure 4), which has been previously found in the marine sponge *Homaxinella* sp. [8].

### 2.4. Agelas citrina

**Figure 5:**
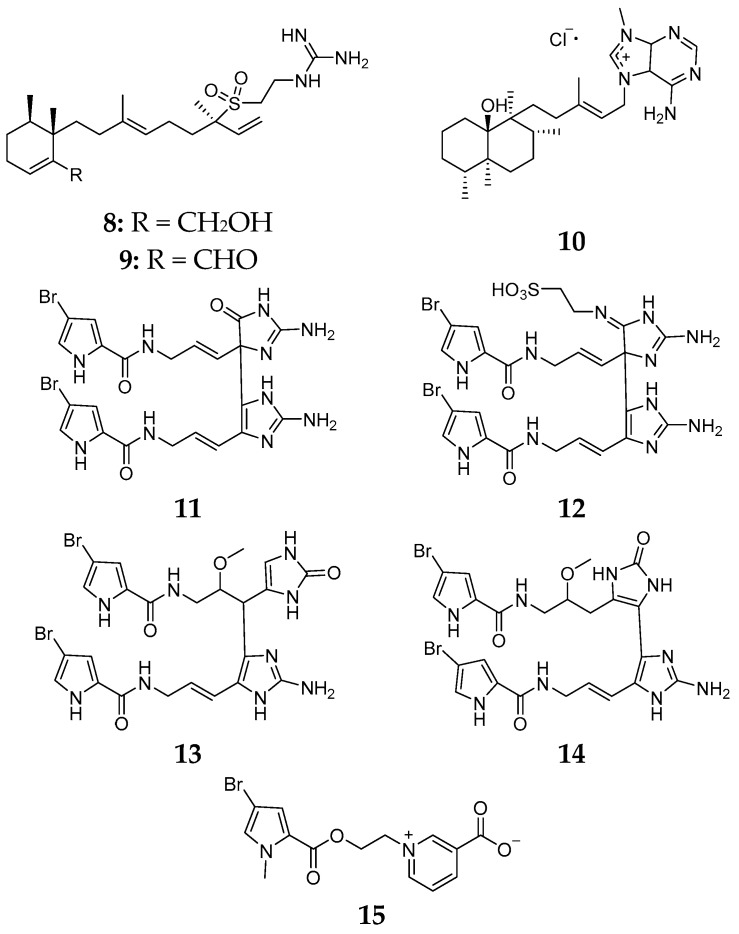
Chemical structures of compounds **8**–**15**.

The Caribbean specimen of *Agelas citrina* was firstly found to yield three new diterpene alkaloids, (−)-agelasidine E (**8**), (−)-agelasidine F (**9**) and agelasine N (**10**) [9]. Latter chemical investigation showed that this sponge also produces four new pyrrole-imidazole alkaloids, citrinamines A–D (**11**–**14**), and one bromopyrrole alkaloid *N*-methylagelongine (**15**) (Figure 5) [10]. Compounds **12**–**14** had antimicrobial activities whereas no inhibitory effect on cell proliferation of mouse fibroblasts was found for **11**–**14**.

### 2.5. Agelas clathrodes

**Figure 6:**
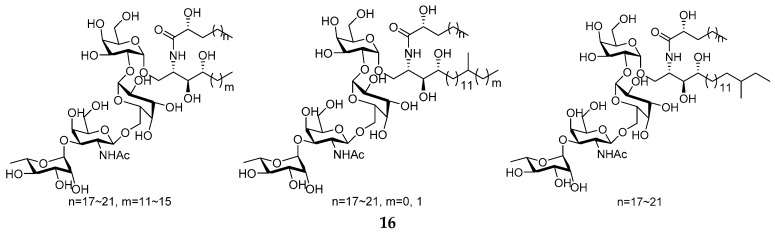

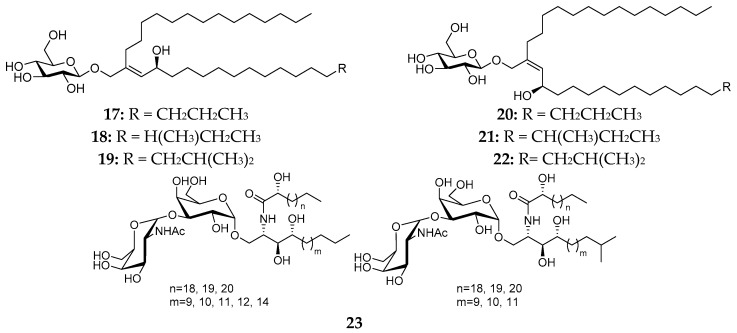
Chemical structures of compounds **16**–**23**.

Marine sponge *Agelas clathrodes* was the excellent producer of secondary metabolites, including glycosphingolipid derivatives (GSLs) and alkaloids. Clarhamnoside (**16**), containing an unusual l-rhamnose unit in the sugar head, was the first rhamnosylated α-galactosylceramide from *A. clathrodes* collected along the coast of Grand Bahamas Island (Sweetings Cay) [11]. The Caribbean sponge *A. clathrodes* could metabolize clathrosides A–C (**17**–**19**) and isoclathrosides A–C (**20**–**22**), which, respectively, belonged to two families of different glycolipids [12]. Compound **23** was also isolated from the Caribbean specimen (Figure 6) [13]. It was noted that all the GSLs from *A. clathrodes* were actually elucidated as mixtures of homologs, which play an important role in therapeutic immunomodulation.

**Figure 7:**
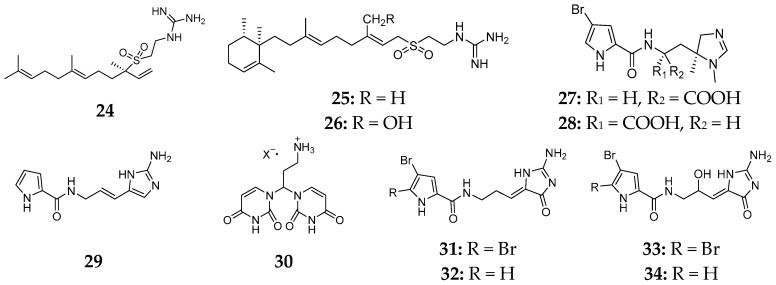
Chemical structures of compounds **24**–**34**.

Six alkaloids, (−)-agelasidine A (**24**), (−)-agelasidine C (**25**), (−)-agelasidine D (**26**), clathramide A (**27**), clathramide B (**28**) and clathrodin (**29**), were detected in the Caribbean sponge *A. clathrodes* (Figure 7). Bioassay results suggested that compound **24** possessed inhibitory effect on *Staphilococcus aureus* but no effect on fungi, while **25** and **26** were shown to have antimicrobial activities against *S. aureus*, *Klebsiella pneumoniae* and *Proteus vulgaris* [14]. In vitro cytotoxic test indicated that **25** and **26** significantly inhibited the growth of CHO-K1 cells with the ED_50_ values of 5.70 and 2.21 μg/mL, respectively. Compound **26** also possessed the inhibition against the growth of *E. coli* and *Hafnia alvei* [15], while **27** and **28** had a moderate antifungal activity against *Aspergillus niger* [16]. Interestingly, compound **29** contained a nonbrominated pyrrole and a guanidine moiety [17]. One specimen of *A. clathrodes* from the South China Sea was shown to produce an ionic compound (**30**), which had weak cytotoxicity against cancer cell lines A549 and SGC7901 with IC_50_ values of 26.5 and 22.7 μg/mL, respectively [18]. Four brominated compounds, dispacamides A–D (**31**–**34**) (Figure 7), were detected not only in *A. clathrodes*, but also in *A. conifera*, *A. dispar* and *A. longissima*, and exhibited antihistamine activity [19,20].

### 2.6. Agelas conifera

**Figure 8:**
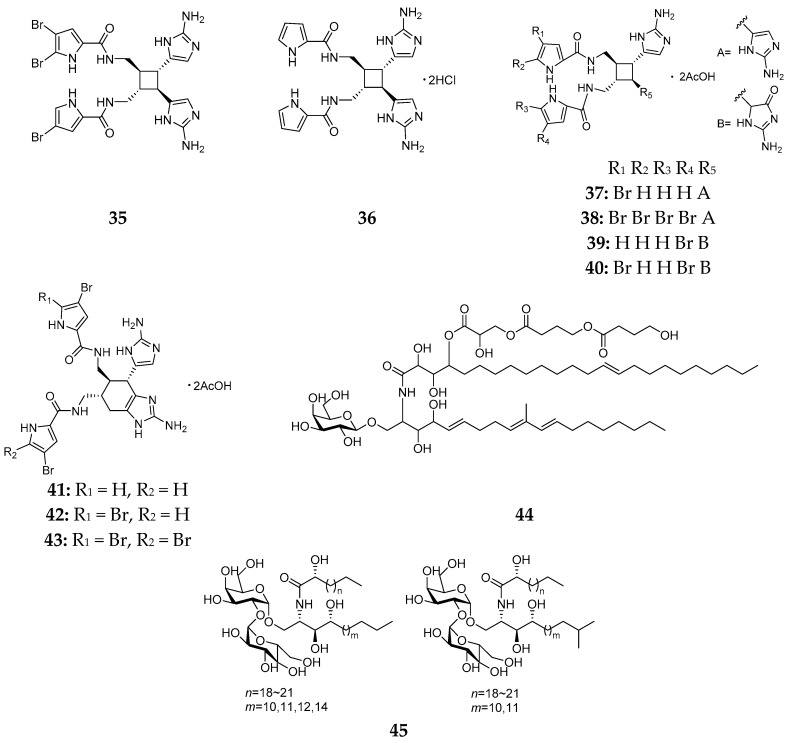
Chemical structures of compounds **35**–**45**.

Chemical study of two specimens of *Agelas conifera* from the Florida Keys and Belize led to the isolation of two new dimeric bromopyrrole alkaloids, bromosceptrin (**35**) and debromosceptrin (**36**), respectively [21,22]. Seven new bromopyrrole metabolites (**37**–**43**) were firstly purified from the Caribbean sponge *A. conifera* [23], but the detailed structure elucidation of ageliferin (**41**), bromoageferin (**42**) and dibromoageliferin (**43**) were established by Kobayashi and his co-workers [24]. Bioassay results indicated that compounds **37**, **41**, **42** and **43** possessed biological activities against *Bacillus subtilis* at 10 μg/disk and **41** and **42** could inhibit the growth of *E. coli* at 10 μg/disk. Using new protein-guided methods by its affinity to proteins within tumor cell proteomes, one unique polyhydroxybutyrated β-GSL coniferoside (**44**), was detected in *A. conifera* derived from Puerto Rico as well as another GSL derivative (**45**) (Figure 8) [25,26].

### 2.7. Agelas dendromorpha

**Figure 9:**
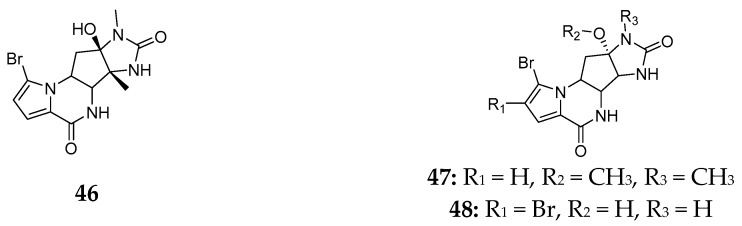
Chemical structures of compounds **46**–**48**.

Natural product analysis of the marine sponge *Agelas dendromorpha* revealed three novel agelastatins (**46**–**48**) with pyrrole-2-imidazole structure. Agelastatin A (**46**) was obtained from the Axinellid specimen grown in the Coral Sea and had strong cytotoxicity [27]. Agelastatins E (**47**) and F (**48**) (Figure 9) purified from the New Caledonian *A. dendromorpha* were shown to exhibit weak cytotoxicity against the KB cell line at 30 μM [28].

### 2.8. Agelas dispar

**Figure 10:**
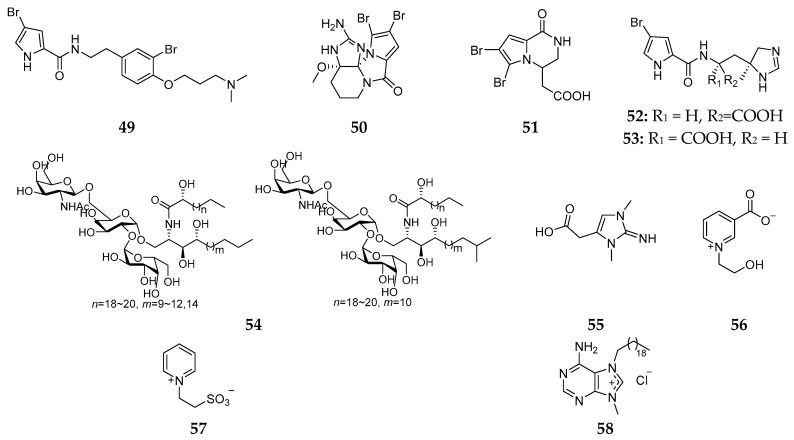
Chemical structures of compounds **49**–**58**.

It is notable that Caribbean *Agelas dispar* harbors a distinct biogeographical bromination trend. Five compounds containing bromine, dispyrin (**49**), dibromoagelaspongin methyl ether (**50**), longamide B (**51**), clathramides C (**52**) and D (**53**), have been found in the Caribbean sponge *A. dispar* [29,30]. Only compound **51** had moderate anti-bacterial activities against *B. subtilis* and *S. aureus* with MIC values of about 50 μg/mL. The GSL derivative (**54**) and betaine alkaloids (**55**–**57**) were detected in the Caribbean *A. dispar* [31,32]. Antibacterial tests indicated that compounds **55** and **56** had the inhibitory activities against *B. subtilis* and *S. aureus* with MIC values ranging from 2.5 to 8.0 μg/mL [32]. The first quaternary derivative of adenine in nature, agelasine (**58**) (Figure 10), was also found in *A. dispar* [33].

### 2.9. Agelas gracilis

**Figure 11:**
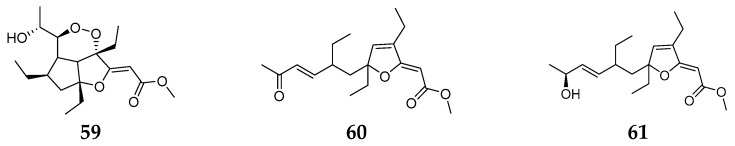
Chemical structures of compounds **59**–**61**.

Bioassay-guided fractionation of the crude extract of the deep-sea sponge *Agelas gracilis* collected in southern Japan afforded three novel antiprotozoan compounds, gracilioethers A–C (**59**–**61**) (Figure 11) [34]. Antimalarial tests showed that these metabolites possessed inhibitory effects on *Plasmodium falciparum* with IC_50_ values of 0.5–10 μg/mL.

### 2.10. Agelas linnaei

**Figure 12:**
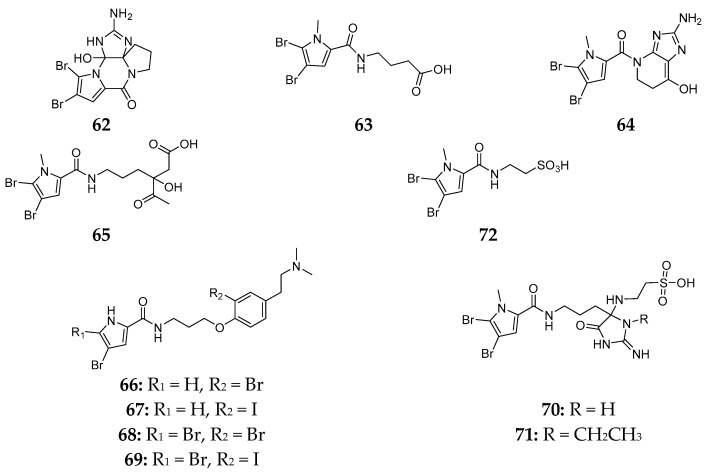
Chemical structures of compounds **62**–**72**.

Eleven novel brominated pyrrole derivatives (**62**–**72**) (Figure 12) were purified from the Indonesian sponge *Agelas linnaei* and compounds **66**–**69** had prominent activities against the murine L1578Y mouse lymphoma cell line with IC_50_ values of 9.55, 9.25, 16.76 and 13.06 μM, respectively [35].

### 2.11. Agelas longissima

**Figure 13:**
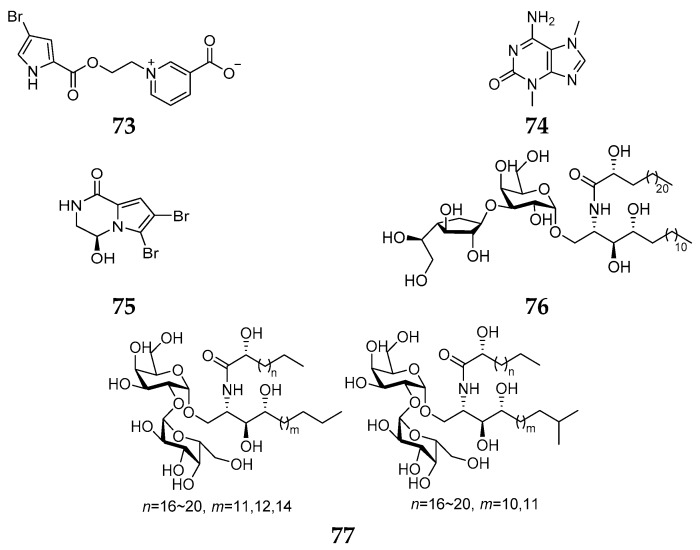
Chemical structures of compounds **73**–**77**.

Five alkaloids (**73**–**77**) (Figure 13) have been isolated from *Agelas longissima* specimens, all of which were collected from the Caribbean Sea. Agelongine (**73**) contained a pyridinium ring instead of the commonly found imidazole nucleus in *Agelas* alkaloids and was shown to be specific to inhibit the agonist 5-hydroxytryptamine (5-HT) [36]. Compound **75** was unique for its unusual pyrrolopiperazine nucleus [37]. Two new GSL analogs (**76** and **77**) were also found in the Caribbean *A. longissima* [38,39].

### 2.12. Agelas mauritiana

**Figure 14:**
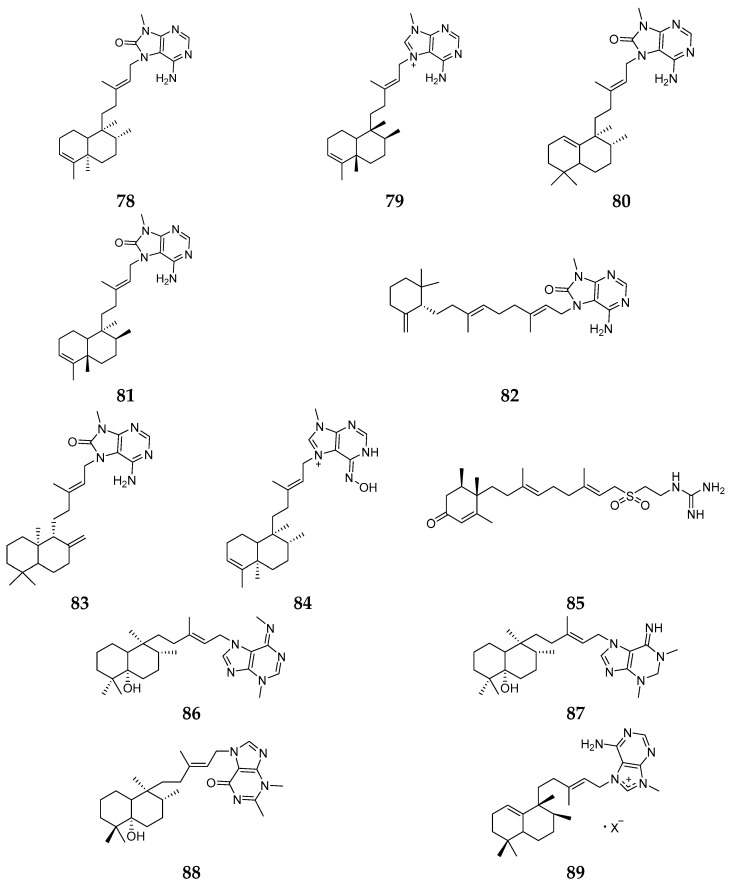

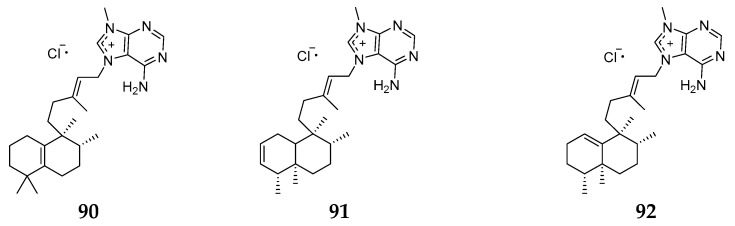
Chemical structures of compounds **78**–**92**.

*Agelas mauritiana* is one of the most fruitful producers of secondary metabolites among all *Agelas* species. Thirty-five compounds (**78**–**112**) have been isolated and identified from *A. mauritiana*, including terpenoids, pyrrole derivatives, GSLs, carotenoids and other alkaloids. Specimens of *A. mauritiana* collected from the South China Sea were found to metabolize eight terpenoids (**78**–**85**) [40,41]. Compound **79** possessed inhibitory effects on *S. aureus* with MIC_90_ value of 1–8 μg/mL and activities against PC9, A549, HepG2, MCF-7, and U937 cell lines with IC_50_ values of 4.49–14.41 μM. Compound **84** possessed activities against *C. neoformans*, *S. aureus*, methicillin-resistant *S. aureus* and *Leishmania donovani* with IC_50_/MIC values of 4.96/10.00, 7.21/10.00, 9.20/20.00 and 28.55/33.19 μg/mL, respectively. Agelasimines A (**86**) and B (**87**) and an unusual purino-diterpene (**88**) were purified from Eniwetak *A. mauritiana* and **86** and **87** had inhibitory effect on L1210 leukemia with ED_50_ values of 2.1 and 3.9 nM, respectively. From Pohnpei-derived *A. mauritiana*, epi-agelasine C (**89**) was isolated and shown to have no activity against *S. aureus*, *Vibrio costicola*, *E. coli* and *B. subtilis* [42,43,44]. Chemical analysis of one specimen of *A. auritiana* collected from the Solomon Islands afforded agelasines J (**90**), K (**91**) and L (**92**) (Figure 14), which exhibited moderate activities against *P. falciparum* and low cytotoxicity on MCF-7 cells [45].

**Figure 15:**
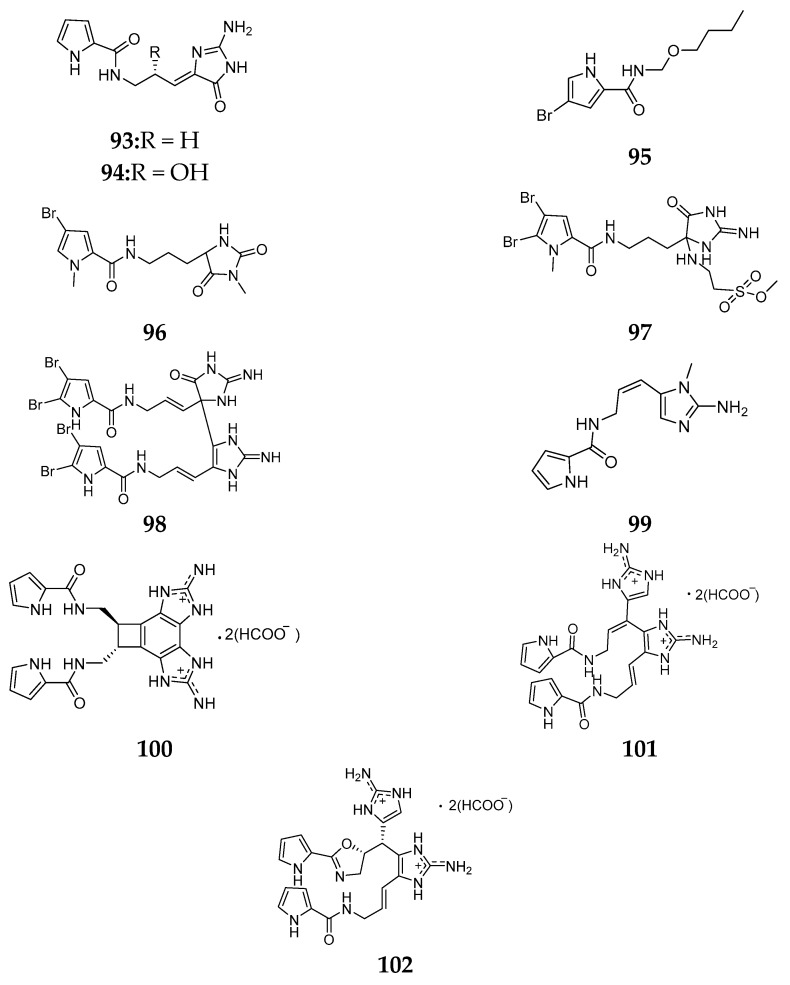
Chemical structures of compounds **93**–**102**.

The same species of *A. mauritiana* grown in different places were found to produce different pyrrole derivatives, such as debromodispacamides B (**93**) and D (**94**) from Solomon Island specimen [46], 4-bromo-*N*-(butoxymethyl)-1*H*-pyrrole-2-carboxamide (**95**) from the South China Sea [41], 5-debromomidpacamide (**96**) from Enewetak Atoll [47], mauritamide A (**97**) from Fiji [48] and mauritiamine (**98**) from Hachijo-jima Island [49]. Compound **98** exhibited inhibitory effect on larval metamorphosis of the barnacle *Balanus amphitrite* with ED_50_ value of 15 μg/mL and moderate antibacterial activity against *Flavobacterium marinotypicum* with the inhibition zone of 10 mm at 10 µg/disk. Interestingly, the Pacific sponge *A. mauritiana* was found to metabolize other pyrroles, including debromokeramadine (**99**), benzosceptrin A (**100**), nagelamide S (**101**) and nagelamide T (**102**) (Figure 15) [50,51].

**Figure 16:**
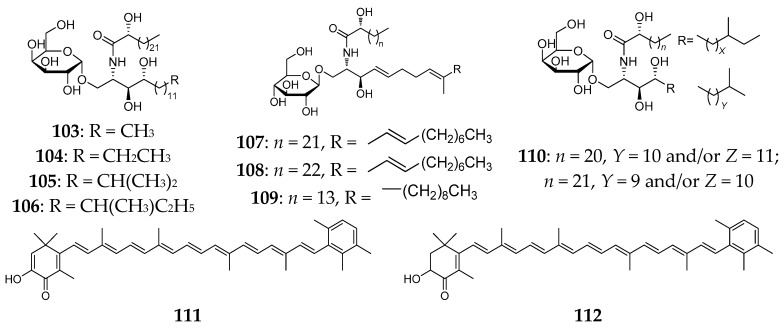
Chemical structures of compounds **103**–**112**.

Agelasphins (**103**–**110**) from the Okinawan *A. mauritiana* were the first example of galactosylceramides containing an α-galactosyl linkage [52,53]. These compounds exhibited high activity with the relative tumor proliferation rate (T/C) ranging from 160% to 190% and 200–400% relative ^3^H-TdR incorporation at <l µg/mL. But no activity was observed against B16 melanoma cells at 20 µg/mL. Two natural carotenoids, isotedanin (**111**) and isoclathriaxanthin (**112**) (Figure 16), were also detected in the specimen of *A. mauritiana* from Kagoshima [54].

### 2.13. Agelas nakamurai

**Figure 17:**
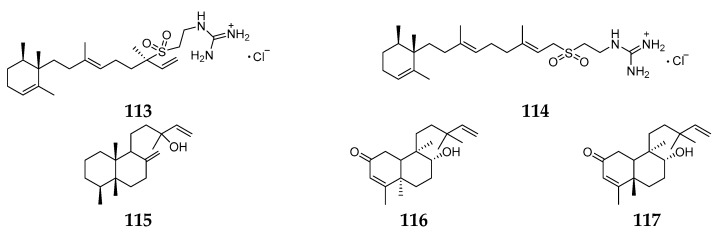

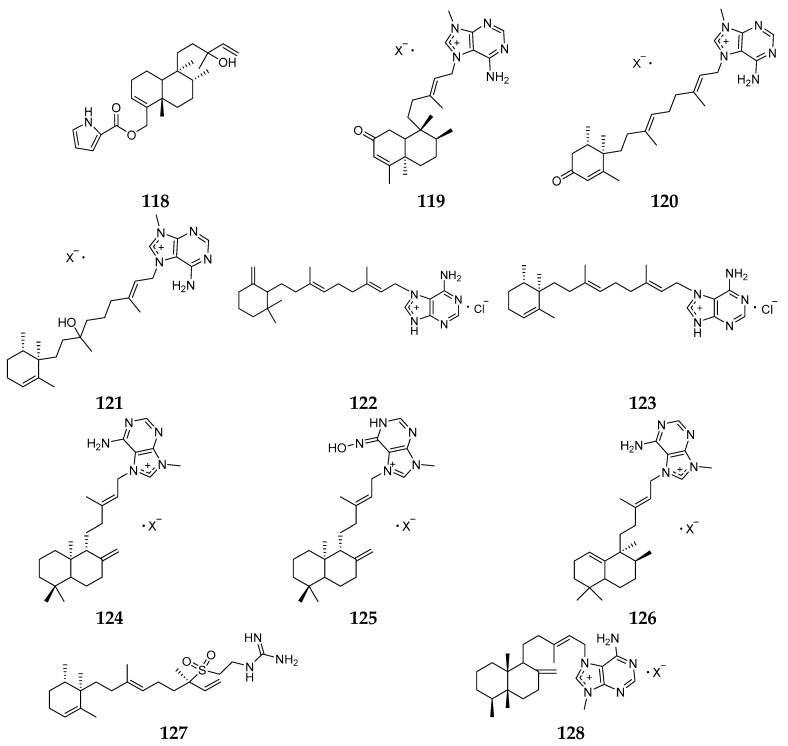
Chemical structures of compounds **113**–**128**.

Thirty-three chemicals have been characterized from *Agelas nakamurai*, including 16 terpenoids and 17 pyrrole alkaloids. The Okinawan *A. nakamurai* seems to occupy the majority of terpenoid compounds, including agelasidines B (**113**) and C (**114**) [55], nakamurols A–D (**115**–**118**) [56], 2-oxoagelasiines A (**119**) and F (**120**), 10-hydro-9-hydroxyagelasine F (**121**) [57], agelasines E (**122**) and F (**123**) [58]. Compounds **113** and **114** were found to have inhibitory effects on the growth of *S. aureus* at 3.3 µg/mL and on contractile responses of smooth muscles. Compounds **119** and **120** showed markedly reduced activity against *Mycobacterium smegmatis*. The Indonesian *A. nakamurai* was found to yield two novel diterpenes, (−)-agelasine D (**124**) and (−)-ageloxime D (**125**). Antibacterial assay revealed **124** could inhibit the growth of *Staphylococcus epidermidis* with a MIC value < 0.0877 µM [35]. Isoagelasine C (**126**) and isoagelasidine B (**127**) were isolated from specimen of the South China Sea and possessed weak cytotoxicities against HL-60, K562 and HCT-116 cell lines with IC_50_ values ranging from 18.4 to 39.2 µM [59]. A new diterpene (**128**) (Figure 17) with a 9-methyladenum moiety produced by the Papua New Guinean *A. nakamurai* Hoshino was shown to be inactive against HIV-1 integrase, *E. coli* and *Pseudomonas aeruginosa* at 12.5 µg/mL [60].

**Figure 18:**
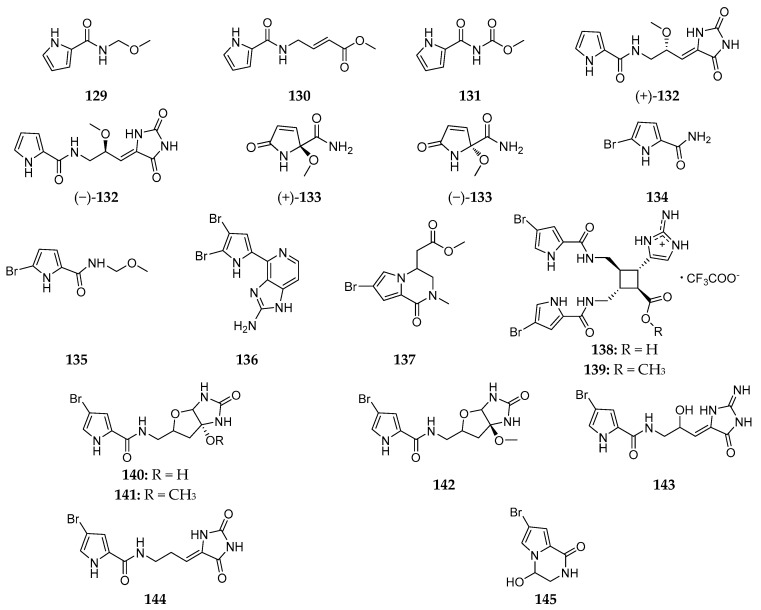
Chemical structures of compounds **129**–**145**.

Five non-brominated pyrrole derivatives, nakamurines A–E (**129**–**133**), were purified from the South China Sea *A. nakamurai* [59,61]. Bioassay results showed that compound **130** had weak inhibition against *Candida albicans* with a MIC value of 60 µg/mL [61]. Bromopyrrole alkaloid was one of the most common secondary metabolites from marine sponges [62]. Two bromopyrrole alkaloids (**134** and **135**) were firstly isolated from the Papua New Guinean *A. nakamurai* in 1998 [60]. Ageladine A (**136**) containing 2-aminoimidazolopyridine was shown to have inhibitory effects on Matrix metalloproteinases-1, -2, -8, -9, -12 and -13 with IC_50_ values of 1.2, 2.0, 0.39, 0.79, 0.33, and 0.47 µg/mL, respectively [63]. Chemical investigation of the Indonesia *A. nakamurai* afforded longamide C (**137**) [35]. Nakamuric acid (**138**) and its methyl ester (**139**) were characterized from the Indopacific specimen and shown to be active against *B. subtilis* [64]. The Okinawan *A. nakamurai* was found to produce six brominated pyrrole derivatives, slagenins A–C (**140**–**142**) and mukanadins A–C (**143**–**145**) (Figure 18), of which **141** and **142** showed inhibitory effect on murine leukemia L1210 cells in vitro with IC_50_ values of 7.5 and 7.0 µg/mL, respectively [65,66].

### 2.14. Agelas nemoechinata

**Figure 19:**
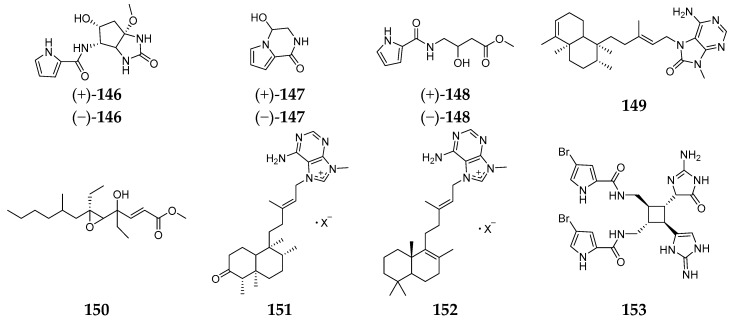
Chemical structures of compounds **146**–**153**.

Nemoechines A–D (**146**–**149**) and nemoechioxide A (**150**) were obtained from the sponge *Agelas* aff. *nemoechinata* collected from the South China Sea. Compounds **146**–**148** had enantiomeric configurations and **146** had an unusual cyclopentene-fused imidazole ring system. Bioassay results suggested that only **149** had cytotoxicity against HL-60 cell lines with an IC_50_ value of 9.9 µM [67]. Two new nemoechine members, nemoechines F (**151**) and G (**152**) possessing *N*-methyladenine, were purified from the South China Sea-derived *A. nemoechinata*. Compound **152** had weak toxicity against Jurkat cell line with an IC_50_ value of 17.1 µM [68]. Oxysceptrin (**153**) (Figure 19) derived from the Okinawan *A. nemoechinata* was a potent actomyosin ATPase activator [69].

### 2.15. Agelas oroides

**Figure 20:**
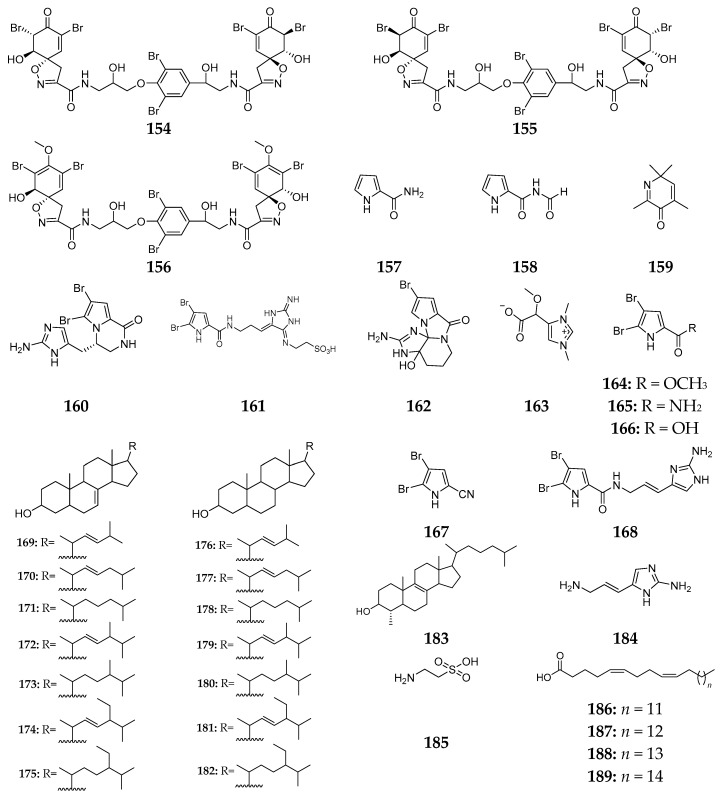
Chemical structures of compounds **154**–**189**.

Thirty-six secondary metabolites (**154**–**189**) (Figure 20) have been isolated from the marine sponge *Agelas oroides*, including pyrrole derivatives, sterols and fatty acids. Chemical investigation of *A. oroides* from the Great Barrier Reef afforded three fistularin-3 derivatives, agelorin A (**154**), agelorin B (**155**) and 11-*epi*-fistularin-3 (**156**). These metabolites exhibited antimicrobial activities against *B. subtilis* and *M. luteus* and **156** had moderate cytotoxicity against breast cancer cells [70]. Later on, two new naturally occurring pyrrole derivatives (**157** and **158**) and 2,4,6,6-tetramethyl-3(6*H*)-pyridone (**159**) were obtained from the same specimen [71,72]. Mediterranean *A. oroides* was shown to produce four novel compounds, cyclooroidin (**160**), taurodispacamide A (**161**), monobromoagelaspongin (**162**) and (−)-equinobetaine B (**163**), of which **161** displayed strong antihistaminic activity [73,74]. Five bromopyrrole alkaloids (**164**–**168**) and fifteen sterols (**169**–**183**) were detected in the sponge *A. oroides* collected in the Bay of Naples [75,76]. Interestingly, one imidazole compound (**184**), taurine (**185**) and some fatty acids (**186**–**189**) were also found in the Northern Aegean Sea-derived specimen [77].

### 2.16. Agelas sceptrum

**Figure 21:**
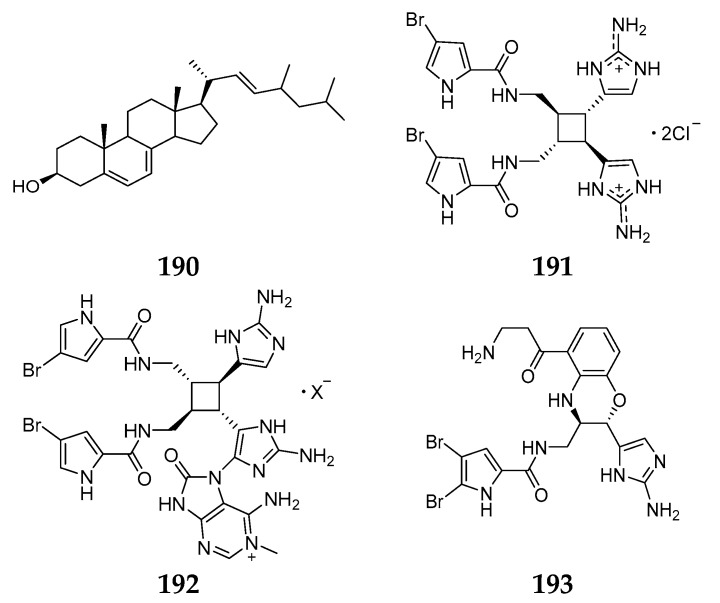
Chemical structures of compounds **190**–**193**.

One novel C_29_ sterol containing the typical nucleus of ergosterol, 26-nor-25-isopropyl-ergosta-5,7,22*E*-trien-3β-ol (**190**), was purified from the Jamaican *A. sceptrum* [78]. Sceptrin (**191**) was obtained from *A. sceptrum* collected at Glover Reef and found to have a broad spectrum of antimicrobial activities against *S. aureus*, *B. subtilis*, *C. albicans*, *Pseudomonas aeruginosa*, *Alternaria* sp. and *Cladosporium cucumerinum* [79]. Chemical study of the sponge from Bahamas afforded two hybrid pyrrole-imidazole alkaloids: 15′-oxoadenosceptrin (**192**) and decarboxyagelamadin C (**193**) (Figure 21) [80].

### 2.17. Agelas schmidtii

**Figure 22:**
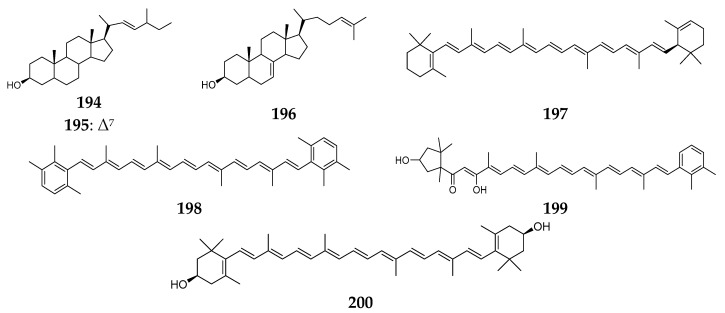
Chemical structures of compounds **194**–**200**.

Three monohydroxyl sterols (**194**–**196**) were isolated from the Caribbean *Agelas schmidtii* [81]. Additionally, four carotenoids named α-carotene (**197**), isorenieratene (**198**), trikentriorhodin (**199**) and zeaxanthin (**200**) (Figure 22) were also derived from this sponge collected from West Indies [82].

### 2.18. Agelas sventres

**Figure 23:**
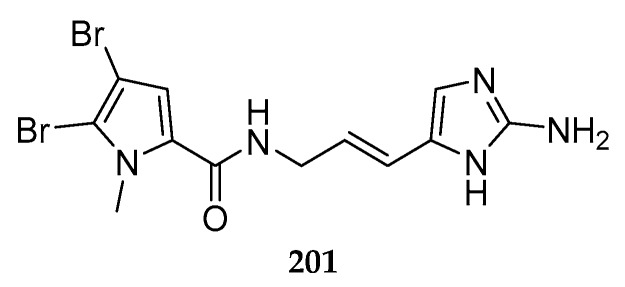
Chemical structure of compounds **201**.

Only one new bromopyrrole alkaloid, sventrin (**201**) (Figure 23), has been purified from the Caribbean sponge *Agelas sventres*. Biological assay showed that this chemical has feeding deterrent activity against omnivorous reef fish [83].

### 2.19. Agelas wiedenmayeri

**Figure 24:**
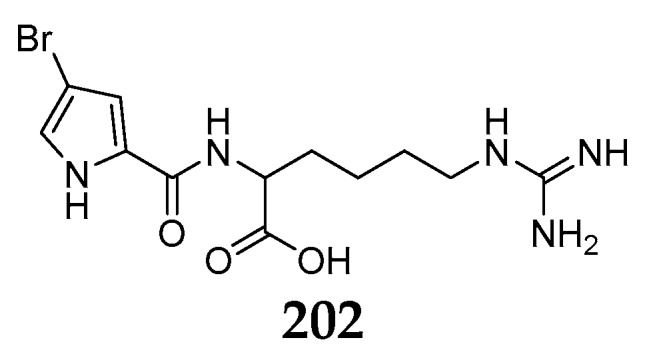
Chemical structure of compounds **202**.

Chemical investigation of *Agelas wiedenmayeri* from Florida Keys afforded one new pyrrole derivative, 4-bromopyrrole-2-carboxyhomoarginine (**202**) (Figure 24), which might be alternatively a biosynthetic precursor of hymenidin/oroidin-related alkaloids [84].

### 2.20. Other Agelas spp.

Eighty-nine secondary metabolites (**203**–**291**) were isolated and chemically identified from unclassified *Agelas* species and assorted into two classes, ionic and non-ionic compounds as below.

#### 2.20.1. Ionic Compounds

**Figure 25:**
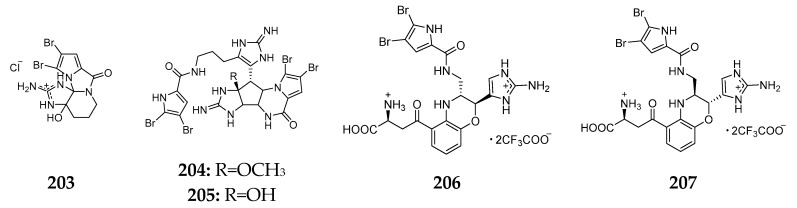

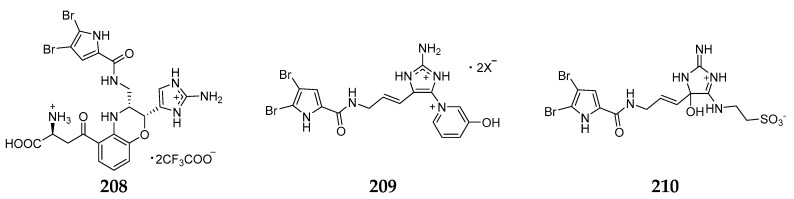
Chemical structures of compounds **203**–**210**.

As described above, ionic compounds are the major secondary metabolites of *Agelas* sponge, which can be grouped in bromine-containing and non-bromine-containing compounds. It is eminent that all ionic brominated metabolites were produced by the Okinawan *Agelas* spp. besides dibromoagelaspongin hydrochloride (**203**) [85]. Agelamadins A (**204**) and B (**205**), possessing an agelastatin-like tetracyclic moiety and an oroidin-like linear moiety, were shown to have antimicrobial activity against *B. subtilis*, *M. luteus* and *C. neoformans* [86]. The same specimen was also found to metabolize agelamadins C–F (**206**–**209**) and tauroacidin E (**210**) (Figure 25), of which **209** was the first occurrence bromopyrrole alkaloid for containing aminoimidazole and pyridinium moieties simultaneously [87,88].

**Figure 26:**
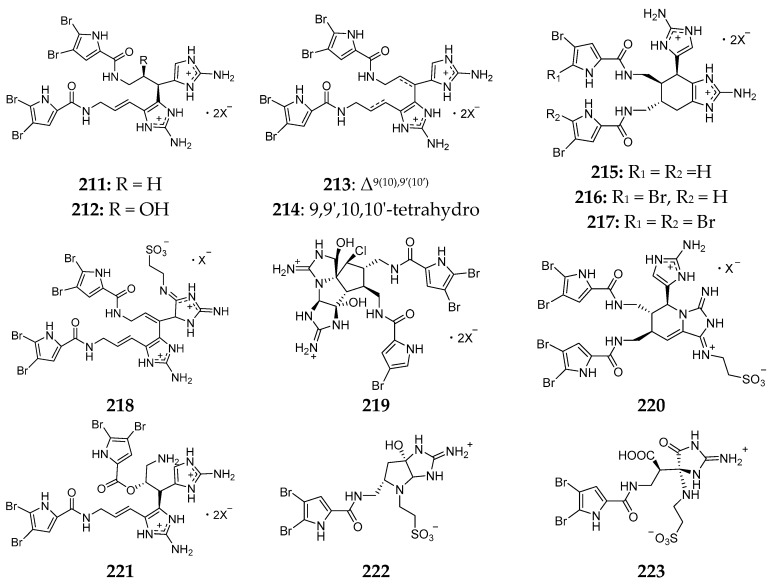

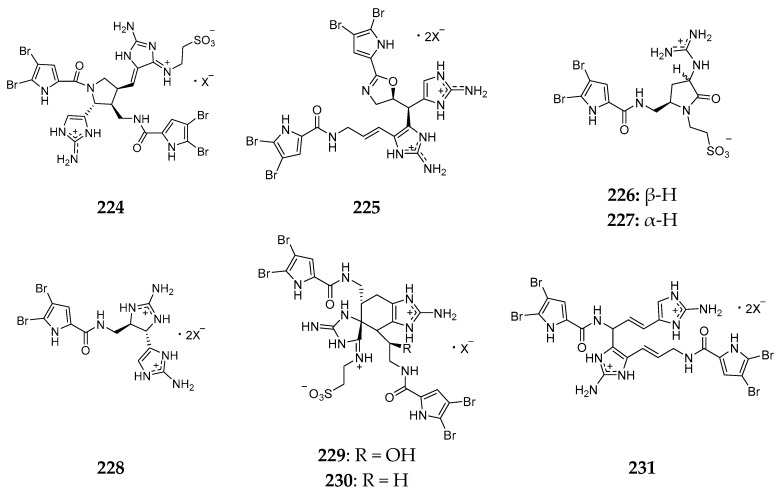
Chemical structures of compounds **211**–**231**.

Twenty-one nagelamides (**211**–**231**) (Figure 26) have been characterized from the Okinawan *Agelas* spp. Nagelamides A–H (**211**–**218**) and O (**219**) were shown to possess antimicrobial activities against *M. luteus*, *B. subtilis* and *E. coli*. Compounds **211**, **217** and **218** were shown to inhibit the growth of protein phosphatase type 2A with IC_50_ values of 48, 13 and 46 µM, respectively [89,90]. Nagelamides K (**220**) and L (**221**) had inhibitory effect on *M. luteus* with a MIC value of 16.7 µg/mL [91]. Bioactivity test uncovered that nagelamides M (**222**) and N (**223**) exhibited inhibition against *A. niger* with the same MIC value of 33.3 µg/mL [92]. Nagelamides Q (**224**) and R (**225**), of which compound **225** possessed an oxazoline ring for the first time, showed antimicrobial activity against *B. subtilis*, *Trichophyton mentagrophytes*, *C. neoformans*, *C. albicans* and *A. niger* [93]. Nagelamides U (**226**) and V (**227**) were the first occurence for a bromopyrrole alkaloid containing a γ-lactam ring with an *N*-ethanesulfonic acid and guanidino moieties, while nagelamide W (**228**) was the first monomeric bromopyrrole alkaloid with two aminoimidazole moieties in the molecule. Compounds **226** and **228** could inhibit against *C. albicans* with the same IC_50_ value of 4 µg/mL [94]. Nagelamides X (**229**) and Y (**230**) were unique for their novel tricyclic skeleton consisting of spiro-bonded tetrahydrobenzaminoimidazole and aminoimidazolidine moieties. In addition, nagelamide Z (**231**) was the first example for dimeric bromopyrrole alkaloid involving the C-8 position in dimerization and displayed strong antimicrobial activity against *C. albicans* with an IC_50_ value of 0.25 µg/mL [95].

**Figure 27:**
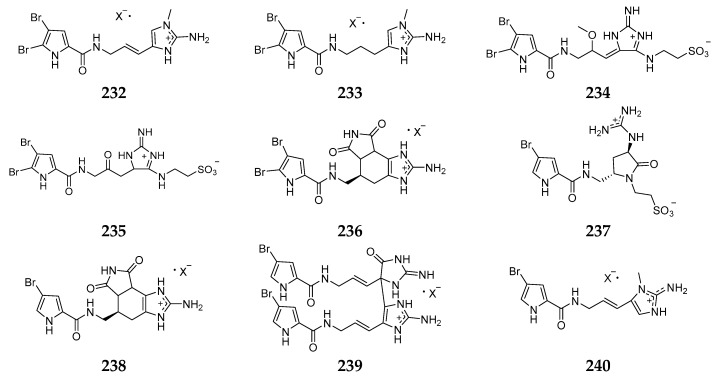

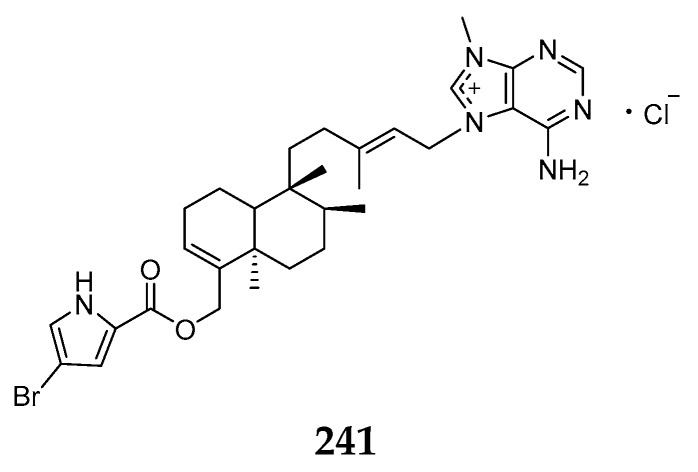
Chemical structures of compounds **232**–**241**.

Eight new bromopyrrole alkaloids, 2-bromokeramadine (**232**), 2-bromo-9,10-dihydrokeramadine (**233**), tauroacidins C (**234**) and D (**235**), mukanadin G (**236**), 2-debromonagelamides U (**237**) and G (**238**), 2-debromonagelamide P (**239**), keramadine (**240**) and agelasine G (**241**) (Figure 27) were detected in the Okinawan *Agelas* spp. [96,97,98,99] Antimicrobial tests suggested that compound **236** exhibited inhibitory effects on *C. albicans* and *C. neoformans* with IC_50_ values of 16 and 8 µg/mL, respectively [96]. Compounds **237** and **239** could inhibit the growth of *T. mentagrophytes* with IC_50_ values of 16 and 32 µg/mL, respectively. Cytotoxicity assay revealed that **241** showed toxicity against murine lymphoma L1210 cells in vitro with an IC_50_ value of 3.1 µg/mL [97,99].

**Figure 28:**
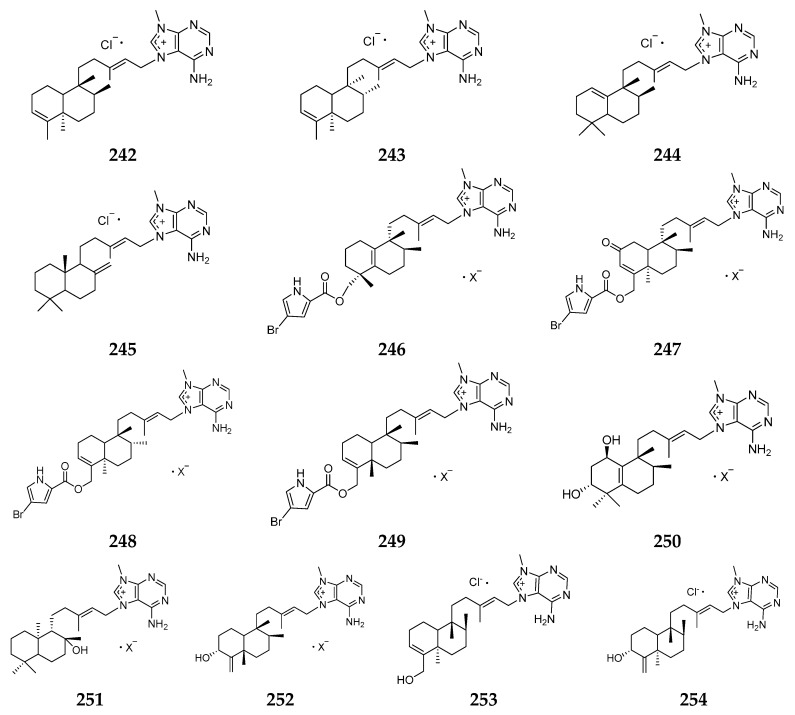

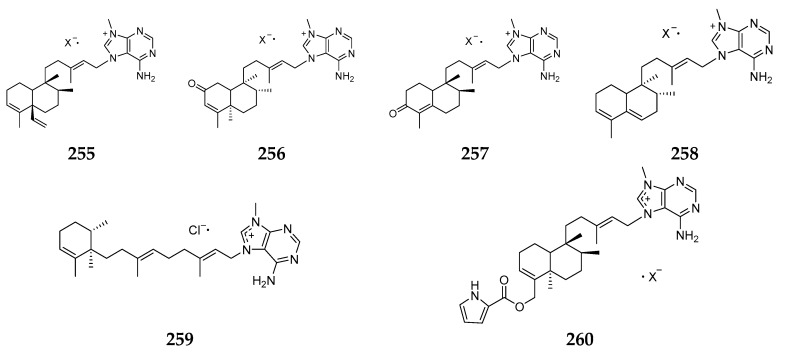
Chemical structures of compounds **242**–**260**.

Nineteen non-bromine-containing ionic compounds have been isolated from unclassified *Agelas* spp., including eleven agalasines (**242**–**252**) from Okinawa [100,101], two agelasines (**253** and **254**) from Yap Island [102], four higher unsaturated 9-*N*-methyladeninium bicyclic diterpenoids (**255**–**258**) from Papua New Guinea [103] and two quarternary 9-methyladenine salts of diterpenes agelines (**259** and **260**) from Argulpelu Reef [104]. Compounds **242**–**245** displayed strong inhibitory effects on Na, K-ATPase and antimicrobial activities [100]. Agelasine M (**255**) exhibited potent activity against *Trypanosoma brucei* [103], while agelines A (**259**) and B (**260**) (Figure 28) showed mild ichthyotoxins and antimicrobial activities [104].

#### 2.20.2. Non-Ionic Compounds

**Figure 29:**
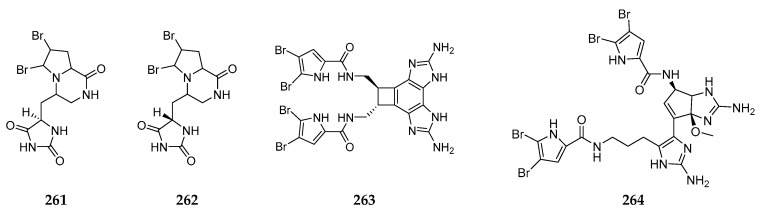

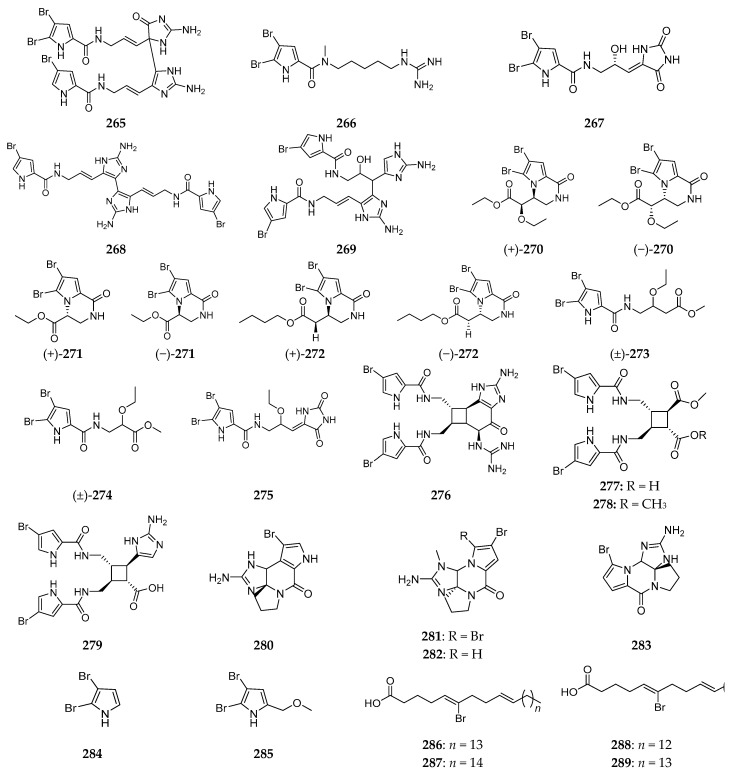
Chemical structures of compounds **261**–**289**.

Since 1983, 29 non-ionic brominated metabolites (**261**–**289**) have been found in some unclassified *Agelas* spp. collected from the Okinawan Sea, the South China Sea, the Caribbean Sea, Papua New Guinea and the Indian Ocean. Agesamides A (**261**) and B (**262**) [105], benzosceptrin C (**263**) [106], nagelamide J (**264**) [107], nagelamide P (**265**), mukanadin E (**266**), mukanadin F (**267**) [92], nagelamide I (**268**) and 2,2’-didebromonagelamide B (**269**) [108] were obtained from the Okinawan specimen. Compound **264** had a cyclopentane ring fused to an amino imidazole ring and exhibited inhibitory effect on *S. aureus* and *C. neoformans* with MIC values of 8.35 and 16.7 µg/mL, respectively. Compounds **268** and **269** were inactive against murine lymphoma L1210 and human epidermoid carcinoma KB cells in vitro. Chemical study of an unidentified *Agelas* spp. from the South China Sea afforded ten new non-ionic bromopyrrole derivatives, longamides D–F (**270**–**272**), 3-oxethyl-4-[1-(4,5-dibromopyrrole-2-yl)-formamido]-butanoic acid methyl ester (**273**), 2-oxethyl-3-[1-(4,5-dibromopyrrole-2-yl)-formamido]-methyl propionate (**274**), 9-oxethyl-mukanadin F (**275**) [109], hexazosceptrin (**276**), agelestes A (**277**) and B (**278**) and (9*S*, 10*R*, 9’*S*, 10’*R*)-nakamuric acid (**279**) [110]. Inspiringly, bioassay results revealed that (+)-**270**, (−)-**271** and (+)-**272** had significant antimicrobial activity against *C. albicans* with MIC values of 80, 20 and 140 µM, respectively. Monobromoisophakellin (**280**) was isolated from the Caribbean *Agelas* sp. and shown to possess antifeedant activity against *Thalassoma bifasciatum* [111]. Chemical investigation of *Agelas* sponges from Wewak and Indonesian sea respectively led to the isolation of two phakellin alkaloids (**281**,**282**) and 5-bromophakelline (**283**) [112,113]. In addition, 2,3-dibromopyrrole (**284**) and 2,3-dibromo-5-methoxymethylpyrrole (**285**) belonging to non-ionic bromopyrrole alkaloid were purified from the marine sponge *Agelas* sp. [114]. Apart from alkaloids, four new brominated phospholipid fatty acids (**286**–**289**) (Figure 29) were also detected in the Caribbean *Agelas* spp. [115].

**Figure 30:**
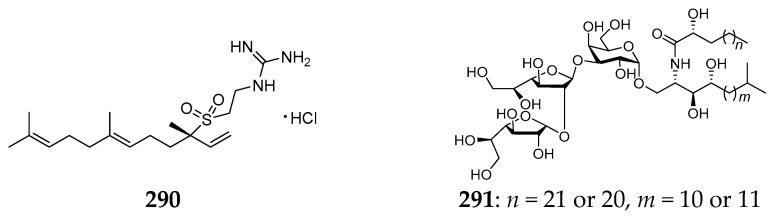
Chemical structures of compounds **290** and **291**.

Only two non-ionic metabolites without bromine, agelasidine A (**290**) and agelagalastatin (**291**) (Figure 30), have been detected in two unclassified specimens of *Agelas* sp. Compound **290** was the first marine natural substance possessing sulfone and guanidine units purified from the Okinawan sample and showed antispasmodic activity [116]. It was notable that compound **24** from the Caribbean *A. clathrodes* is the optimal isomer of **290**. Compound **291** was a new GSL derived from *Agelas* sp. collected in Papua New Guinea and found to exhibit significant in vitro activity against human cancer cell lines with lung NCI-H460 GI_50_ 0.77 µg/mL to ovary OVCAR-3 GI_50_ 2.8 µg/mL [117].

## 3. Conclusions

Many efforts have been devoted to implement chemical investigation of *Agelas* sponges during the past 47 years, from 1971 to 2017. Meanwhile, great achievements have been made on chemical diversity of their secondary metabolites. *Agelas* sponges are widely distributed in the ocean, especially in the Okinawa Sea, the Caribbean Sea and the South China Sea. Deep ocean technologies for specimen collecting should be used to search more unknown species of *Agelas* sponges, such as manned and remotely operated underwater vehicles. Advanced separation methodologies should be deployed to explore more bioactive secondary metabolites of these sponges, such as UPLC-MS, metabolomics approach [74]. Furthermore, special attention should be paid to symbiotic microorganisms of *Agelas* sponges owing to the fact that a great number of therapeutic agents of marine sponges are biosynthesized by their symbiotic microbes [118]. By a combination of gene engineering, pathway reconstructing, enzyme engineering and metabolic networks, these microbes can be modified to produce more novel chemicals containing enhanced structural features or a large quantity of known valuable compounds for pharmaceutical production.

## Figures and Tables

**Table 1:** *Agelas*-derived secondary metabolites.

Organism	Locality	Secondary Metabolite	References
*Agelas axifera*	the Republic of Palau	axistatins 1 (**1**), 2 (**2**), 3 (**3**)	[4]
*A. cerebrum*	Caribbean	5-bromopyrrole-2-carboxylic acid (**4**), 4-bromopyrrole-2-carboxylic acid (**5**), 3,4-bromopyrrole-2-carboxylic acid (**6**)	[6]
*A. ceylonica*	the Mandapam coast	hanishin (**7**)	[7]
*A. citrina*	Caribbean	(−)-agelasidine E (**8**), (−)-agelasidine F (**9**), agelasine N (**10**), citrinamines A–D (**11**–**14**), *N*-methylagelongine (**15**)	[9,10]
*A. clathrodes*	Grand Bahamas Island	clarhamnoside (**16**)	[11]
	Caribbean	clathrosides A–C (**17**–**19**), isoclathrosides A–C (**20**–**22**), glycosphingolipid (**23**), (−)-agelasidine A (**24**), (−)-agelasidine C (**25**), (−)-agelasidine D (**26**), clathramides A (**27**) and B (**28**), clathrodin (**29**), dispacamides A–D (**31**–**34**)	[12,13,14,15,16,17,19,20]
	South China Sea	3,3-bis(uracil-l-yl)-propan-1-aminium (**30**)	[18]
*A. conifera*	Florida Keys	bromosceptrin (**35**)	[21]
	Belize	debromosceptrin (**36**)	[22]
	Caribbean	bromopyrroles (**37**–**43**), glycosphingolipid (**45**)	[23,24,26]
	Puerto Rico	coniferoside (**44**)	[25]
*A. dendromorpha*	the Coral Sea	agelastatin A (**46**)	[27]
	the New Caledonia	agelastatins E (**47**) and F (**48**)	[28]
*A. dispar*	Caribbean	dispyrin (**49**), dibromoagelaspongin methyl ether (**50**), longamide B (**51**), clathramides C (**52**) and D (**53**), aminozooanemonin (**55**), pyridinebetaines A (**56**) and B (**57**)	[29,30,32]
	San Salvador Island	triglycosylceramide (**54**)	[31]
		agelasine (**58**)	[33]
*A. gracilis*	South Japan	gracilioethers A–C (**59**–**61**)	[34]
*A. linnaei*	Indonesia	brominated pyrrole derivatives (**62**–**72**)	[35]
*A. longissima*	Caribbean	agelongine (**73**), 3,7-dimethylisoguanine (**74**), longamide (**75**), glycosphingolipids (**76** and **77**)	[36,37,38,39]
*A. mauritiana*	South China Sea	(−)-80-oxo-agelasine B (**78**), (+)-agelasine B (**79**), (+)-8’-oxo-agelasine C (**80**), agelasine V (**81**), (+)-8’-oxo-agelasine E (**82**), 8’-oxo-agelasine D (**83**), ageloxime B (**84**), (+)-2-oxo-agelasidine C (**85**), 4-bromo-*N*-(butoxymethyl)-1*H*-pyrrole-2-carboxamide (**95**)	[40,41]
	Enewetak	agelasimine A (**86**), agelasimine B (**87**), purino-diterpene (**88**), 5-debromomidpacamide (**96**)	[42,43,47]
	Pohnpei	*epi*-agelasine C (**89**)	[44]
	Solomon Islands	agelasines J (**90**), K (**91**) and L (**92**), debromodispacamides B (**93**) and D (**94**)	[45,46]
	Fiji	mauritamide A (**97**)	[48]
	Hachijo-jima Island	mauritiamine (**98**)	[49]
	the Pacific sea	ebromokeramadine (**99**), benzosceptrin A (**100**), nagelamides S (**101**) and T (**102**)	[50,51]
	Okinawa	agelasphins (**103**–**110**)	[52,53]
	Kagoshima	isotedanin (**111**), isoclathriaxanthin (**112**)	[54]
*A. nakamurai*	Okinawa	agelasidines B (**113**) and C (**114**), nakamurols A–D (**115**–**118**), 2-oxoagelasiines A (**119**) and F (**120**), 10-hydro-9-hydroxyagelasine F (**121**), agelasines E (**122**) and F (**123**), slagenins A–C (**140**–**142**), mukanadins A–C (**143**–**145**)	[55,56,57,58,65,66]
	Indonesia	(−)-agelasine D (**124**), (−)-ageloxime D (**125**)	[35]
	South China Sea	isoagelasine C (**126**), isoagelasidine B (**127**)	[59]
	Papua New Guinea	diterpene (**128**), bromopyrrole alkaloids (**134** and **135**)	[60]
	South China Sea	nakamurines A–E (**129**–**133**)	[59,61]
	Japan	ageladine A (**136**)	[63]
	Indonesia	longamide C (**137**)	[35]
	Indopacific	nakamuric acid (**138**) and its methyl ester (**139**)	[64]
*A. nemoechinata*	South China Sea	nemoechines A–D (**146**–**149**), nemoechioxide A (**150**), nemoechines F (**151**) and G (**152**)	[67,68]
	Okinawa	oxysceptrin (**153**)	[69]
*A. oroides*	the Great Barrier Reef	agelorin A (**154**), agelorin B (**155**), 11-*epi*-fistularin-3 (**156**), pyrrole-2-carboxamide (**157**), *N*-formyl-pymole-2-carboxamid (**158**), 2,4,6,6-tetramethyl-3(6*H*)-pyridone (**159**)	[70,71,72]
	Mediterranea Sea	cyclooroidin (**160**) and taurodispacamide A (**161**), monobromoagelaspongin (**162**), (−)-equinobetaine B (**163**)	[73,74]
	Naples	bromopyrroles (**164**–**168**), sterols (**169**–**183**)	[75,76]
	the Northern Aegean Sea	3-amino-1-(2-aminoimidazoyl)-prop-1-ene (**184**), taurine (**185**), fatty acid mixtures (**186**–**189**)	[77]
*A. sceptrum*	Jamaica	26-nor-25-isopropyl-ergosta-5,7,22 *E*-trien-3β-ol (**190**)	[78]
	Belize	sceptrin (**191**)	[79]
	Bahamas	15′-oxoadenosceptrin (**192**), decarboxyagelamadin C (**193**)	[80]
*A. schmidtii*	Caribbean	monohydroxyl sterols (**194**–**196**)	[81]
	West Indies	α-carotene (**197**), isorenieratene (**198**), trikentriorhodin (**199**) and zeaxanthin (**200**)	[82]
*A. sventres*	Caribbean	sventrin (**201**)	[83]
*A. wiedenmayeri*	Florida Keys	4-bromopyrrole-2-carboxyhomoarginine (**202**)	[84]
Unclassified* Agelas *sp.	No record	dibromoagelaspongin hydrochloride (**203**)	[85]
	Okinawa	agelamadins A (**204**) and B (**205**), agelamadins C–F (**206**–**209**), tauroacidin E (**210**), nagelamides A–H (**211**–**218**), nagelamides K–O (**219**–**223**), nagelamides Q (**224**) and R (**225**), nagelamides U–Z (**226**–**231**), 2-bromokeramadine (**232**), 2-bromo-9,10-dihydrokeramadine (**233**), tauroacidins C (**234**) and D (**235**), mukanadin G (**236**), 2-debromonagelamides U (**237**) and G (**238**), 2-debromonagelamide P (**239**), keramadine (**240**), agelasine G (**241**), agelasines A–D (**242**–**245**), agelasines O–U (**246**–**252**), agesamides A (**261**) and B (**262**), benzosceptrin C (**263**), nagelamides J (**264**) and P (**265**), mukanadins E (**266**) and F (**267**), nagelamide I (**268**), 2,2’-didebromonagelamide B (**269**), agelasidine A (**290**)	[86,87,88,89,90,91,92,93,94,95,96,97,98,99,100,101,105,106,107,108,116]
	Yap Island	agelasines H (**253**) and I (**254**)	[102]
	Papua New Guinea	agelasine M (**255**), 2-oxo-agelasine B (**256**), gelasines A (**257**) and B (**258**), (−)-7-*N*-methyldibromophakellin (**281**), (−)-7-*N*-methylmonobromophakellin (**282**), agelagalastatin (**291**)	[103,112,117]
	Palau Island	agelines A (**259**) and B (**260**)	[104]
	South China Sea	longamides D–F (**270**–**272**), 3-oxethyl-4-[1-(4,5-dibromopyrrole-2-yl)-formamido]-butanoic acid methyl ester (**273**), 2-oxethyl-3-[1-(4,5-dibromopyrrole-2-yl)-formamido]-methyl propionate (**274**), 9-oxethyl-mukanadin F (**275**), hexazosceptrin (**276**), agelestes A (**277**) and B (**278**) and (9*S*,10*R*,9’*S*,10’*R*)-nakamuric acid (**279**)	[109,110]
	Caribbean Sea	monobromoisophakellin (**280**), brominated phospholipid fatty acids (**286**–**289**)	[111,115]
	Indonesia	5-bromophakelline (**283**)	[113]
	No record	2,3-dibromopyrrole (**284**) and 2,3-dibromo-5-methoxymethylpyrrole (**285**)	[114]
